# Analysis of the demand for mental health consultation and liaison programmes: a comprehensive view.

**DOI:** 10.1192/j.eurpsy.2024.550

**Published:** 2024-08-27

**Authors:** F. Garcia Lazaro, F. Gotor Sanchez-Luengo

**Affiliations:** Hospital Universitario Virgen del Rocio, Sevilla, Spain

## Abstract

**Introduction:**

The mental health consultation and liaison programme plays a crucial role in comprehensive medical care by addressing psychiatric co-morbidities in hospitalised patients.

**Objectives:**

The aim of this study is to analyse demand and assess referral patterns to the mental health consultation and liaison programme in order to identify areas for improvement and optimise the provision of care.

**Methods:**

A descriptive cross-sectional study was conducted by analysing records of referrals to the mental health consultation and liaison programme over a one-year period. Demographic data, origin of demand, type of request, episodic diagnosis, psychiatric diagnoses, follow-up and discharge referral were collected.

**Results:**

A total of 1180 referrals to the mental health consultation and liaison programme were reviewed. Most of the episodic diagnoses were related to anxious-depressive symptomatology, acute stress reaction and acute confusional syndrome. The majority of patients followed up did not require further referral to mental health facilities.

**Conclusions:**

Analysis of the demand for the mental health consultation and liaison programme helps us to optimise care on psychiatric co-morbidities. These results support the importance of integrated care that addresses both medical and psychiatric aspects of inpatient health. Strategies to improve collaboration between different services should be implemented to ensure optimal care and provide a holistic approach.

**Disclosure of Interest:**

None Declared

